# Combining DNA damaging therapeutics with immunotherapy: more haste, less speed

**DOI:** 10.1038/bjc.2017.376

**Published:** 2017-11-09

**Authors:** Jessica S Brown, Raghav Sundar, Juanita Lopez

**Affiliations:** 1 Royal Marsden NHS Foundation Trust, Downs Road, London SM2 5PT, UK; 2 Department of Haematology-Oncology, National University Health System, Singapore; 3 The Institute of Cancer Research, London SM2 5NG, UK

**Keywords:** DNA damage, immunotherapy, immune checkpoint inhibitors, PD-1, PD-L1, CTLA-4, immunogenic cell death, neoantigens

## Abstract

The idea that chemotherapy can be used in combination with immunotherapy may seem somewhat counterproductive, as it can theoretically eliminate the immune cells needed for antitumour immunity. However, much preclinical work has now demonstrated that in addition to direct cytotoxic effects on cancer cells, a proportion of DNA damaging agents may actually promote immunogenic cell death, alter the inflammatory milieu of the tumour microenvironment and/or stimulate neoantigen production, thereby activating an antitumour immune response. Some notable combinations have now moved forward into the clinic, showing promise in phase I–III trials, whereas others have proven toxic, and challenging to deliver. In this review, we discuss the emerging data of how DNA damaging agents can enhance the immunogenic properties of malignant cells, focussing especially on immunogenic cell death, and the expansion of neoantigen repertoires. We discuss how best to strategically combine DNA damaging therapeutics with immunotherapy, and the challenges of successfully delivering these combination regimens to patients. With an overwhelming number of chemotherapy/immunotherapy combination trials in process, clear hypothesis-driven trials are needed to refine the choice of combinations, and determine the timing and sequencing of agents in order to stimulate antitumour immunological memory and improve maintained durable response rates, with minimal toxicity.

Without doubt, a subset of cancer patients have experienced tremendous benefit from the clinical implementation of immune checkpoint inhibitors, and naturally therefore attention is now focussing on mechanisms by which primary and secondary resistance can be overcome. This has largely been approached through therapeutic combination strategies and the recent publication of Keynote 021 (Langer *et al*, 2016) demonstrates that such combinations are safe and can be more effective than chemotherapy alone. Keynote 021 is the first trial to publish a benefit of immune checkpoint inhibition with a PD-1 inhibitor in combination with chemotherapy over chemotherapy alone, in this case as first-line treatment for patients with non-small-cell lung cancer (NSCLC). Whether this represents a synergistic interaction between chemotherapy and immune checkpoint inhibition rather than an additive effect has yet to be established, but there is a great deal of evidence in support of combining DNA damaging agents with immune modulating drugs.

In this review we will discuss the preclinical rationale for combining immune checkpoint inhibitors with DNA damaging agents. We will summarise the clinical experience with such combinations so far and highlight the challenges faced when combining immunotherapy with DNA damaging therapeutics in the clinic.

## Tumour immunosurveillance

The host immune system actively protects itself against tumour development, and evasion of cancer immunosurveillance through both local immunosuppression within the tumour microenvironment (TME) and emergence of an immunoevasive phenotype through immunoediting is an emerging hallmark of many solid tumours (Schreiber *et al*, 2011; Joyce and Fearon, 2015).

The existence of tumour-specific antigens, which may include the products of mutated genes (neoantigens), or proteins expressed only in the tumour and not in normal tissues for example, has been known for some time (Schreiber *et al*, 2011). Immunoediting involves the elimination of immunologically foreign tumour cells through the activity of the innate and adaptive immune systems (Schreiber *et al*, 2011). This may serve to eradicate the tumour entirely, or as a result of tumour heterogeneity, selectively destroy highly immunogenic tumour cells that, over time, results in a tumour largely composed of poorly immunogenic and immunoevasive cells (Schreiber *et al*, 2011).

An effective adaptive immune response requires that: cytotoxic T cells (CD8+) are sufficiently activated, that tumour-specific T cells navigate to the tumour; extravasate from the vasculature and cross the TME, before recognising and responding to their target antigen. The mere presence of tumour-specific cytotoxic T cells is therefore not sufficient for immune-mediated tumour cell death, and extrinsic to the tumour cells themselves, early adoption of an immunosuppressive TME enables tumours to develop in what are essentially immune-privileged sites (Joyce and Fearon, 2015). This immunosuppression is essential in evading immunosurveillance and is achieved through a number of overlapping mechanisms (Box 1).

## Immune checkpoint inhibitors

Most recently, clinical focus has centred on the T-cell immune checkpoint inhibitors. T-cell activation requires the interaction between the T-cell receptor (TCR) and major histocompatability complex (MHC) bound to tumour-derived peptide on the antigen-presenting cell (APC), alongside costimulation provided by interaction between CD28 on the T cell and B7 ligand on the APC (Sharma and Allison, 2015). Immune checkpoint inhibitors release the physiological suppression of T-cell activation.

CTLA-4 is a T-cell inhibitory receptor that competitively antagonises the costimulatory interaction between CD28 and B7 ligand. Expression of CTLA-4 on T cells is induced following T-cell activation where it functions to attenuate and eventually terminate T-cell activation (Sharma and Allison, 2015). Anti-CTLA-4 monoclonal antibody (mAb) treatment therefore results in persistent T-cell activation and subsequent trafficking of T cells to sources of antigen. Its use has been associated with an increased number of new tumour-specific CD8+ T cells in patients with melanoma, suggesting that it broadens the immune response, rather than just stimulating it (Kvistborg *et al*, 2014). It has also been shown to deplete regulatory T (Treg) cells in the tumour microenvironment (Simpson *et al*, 2013). Ipilumumab, an antibody against human CTLA-4, was licenced in 2011 after showing significant clinical benefit in patients with metastatic melanoma (Hodi *et al*, 2010) (Table 1).

The second class of immune checkpoint inhibitors that are transforming standard of care across a range of tumour types are inhibitors of programmed cell death-1/programmed cell death ligand-1 (PD-1/PD-L1) signalling. Similar to CTLA-4, PD-1 is expressed on activated T cells but, unlike CTLA-4, PD-1 interaction with its most studied ligands PD-L1 or PD-L2 inhibits T-cell activation through interfering with downstream TCR signalling (Patsoukis *et al*, 2012). Interferon-*γ* (IFN-*γ*), released as a result of T-cell activation, can induce PD-L1 expression on a range of cell types, including nonlymphoid tissue such as epithelial, endothelial and tumour cells (Sharma and Allison, 2015). The expression of PD-L1 in tumours is therefore driven by the presence of activated T cells in the TME and upregulation of PD-L1 in tumours is an effective means of evasion of immunosurveillance. The expression of PD-L2, although also being cytokine induced, is restricted to macrophages and dentritic cells (Greenwald *et al*, 2005).

Current thinking is that CTLA-4 is required for limiting T-cell activity centrally within the lymphovascular system (central immune tolerance), whereas PD-1 signalling plays a more prominent role during later stages of the immune response and is required for the inhibition of T-cell signalling in peripheral tissues (peripheral tolerance). Pembrolizumab and nivolumab, both PD-1 inhibitors, as well as atezolizumab and durvalumab, a PD-L1 inhibitor, all now hold licences for clinical use (Table 1).

## Effects of DNA damaging agents on the immune system

DNA damage arises either due to cellular exposure to exogenous sources of damaging agents such as chemotherapy or because of a failure to repair endogenous DNA damage in cells (Jackson and Bartek, 2009). DNA damage itself can take many different forms depending on the mechanism of action of the agent used (Box 2), with DNA double-strand breaks (DSBs) considered to be the most cytotoxic to cancer cells (Brown *et al*, 2017). DNA damaging agents are widely used as standard of care treatment across a range of tumour types. Inhibiting the repair of endogenous or exogenous DNA damage is also an attractive anticancer strategy and several different DNA repair inhibitors are in clinical development (Brown *et al*, 2017). Although in this review we will focus on the immunomodulatory effects of chemotherapy, as well as the newer DNA repair inhibitors, it must be noted that the immune effects of radiotherapy are also well reported and have recently been reviewed elsewhere (Weichselbaum *et al*, 2017).

**Figure 1:**
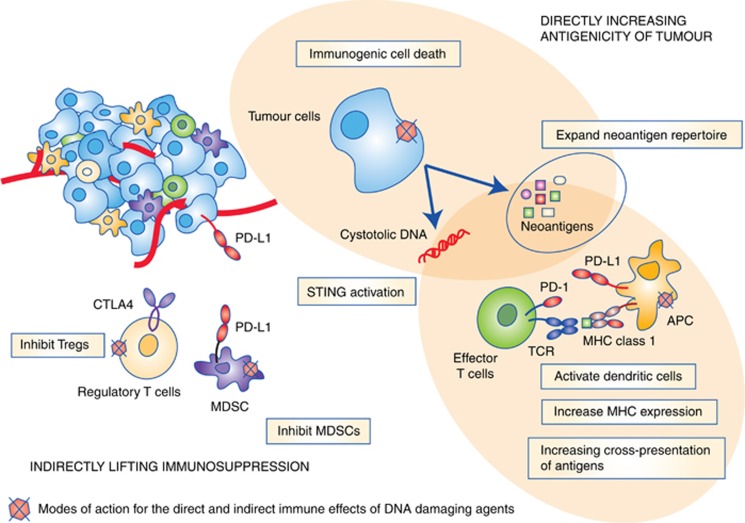
**Mechanisms by which DNA damaging agents affect the immunogenicity of tumours.** See text for details.

Traditionally, chemotherapy has been considered immunosuppressive and several chemotherapeutics, such as methotrexate and cyclophosphamide, are used to treat autoimmune conditions. The choice of DNA damaging therapeutic agent, dose of compound and timing of these combinations is important therefore, not least because many cytotoxic chemotherapeutics have the potential to deplete rapidly dividing immune-cell populations. DNA damaging agents have now been shown to promote immunogenicity in a variety of ways however, many of which have the potential to be exploited in a clinical setting (Figure 1).

### Increasing neoantigen repertoire

Tumour neoantigens are predominantly felt to be the consequences of the genetic alterations accumulated by cancer cells during tumourigenesis. They have been demonstrated to arise from various processes that alter the open reading frame (ORF) sequences in the genome (Hacohen *et al*, 2013). Not only missense mutations, but also fusion transcripts, frameshifts and stop losses can also potentially create altered ORFs (i.e., neoORFS) encoding novel stretches of amino acids that are not present in the normal genome. A significant advantage of increasing neoantigen load is that neoantigens are tumour specific and central T-cell tolerance is therefore not a concern. Neoantigens are however patient specific and stimulating T-cell responses against tumour-specific immunogenic clonal neoantigens is currently not a high-throughput strategy.

There is accumulating evidence to suggest that high mutational load equates with increased antigenicity, however, as directly identifying HLA-bound neoantigens on tumour tissue has yet to be successful, proving this association definitively is difficult, and most studies rely on mathematical predictions of neoantigen load. Tumours vary in the number of somatic mutations they harbour, with melanoma, lung and bladder cancer having on average the highest mutational load (Alexandrov *et al*, 2013) and also showing highest responses to immune checkpoint inhibitors. Within tumour types, high mutational load has been demonstrated to correlate with clinical benefit to PD-1 and CTLA-4 inhibitors in NSCLC and melanoma, respectively (Snyder *et al*, 2014; Rizvi *et al*, 2015). Most studies to date have focussed on the burden of single-nucleotide variants (SNVs) as a measure of mutational load, but the quantity of small insertions and deletions (indels) resulting in frameshift mutations also correlates with checkpoint inhibitor response in melanoma patient cohorts (Turajlic *et al*, 2017), demonstrating that frameshift mutations are also likely to significantly contribute to neoantigen repertoire. In addition, frameshift mutations in microsatellite unstable colorectal cancers have been shown to correlate with the density of CD8+ T-cell infiltrate in tumours (Maby *et al*, 2015).

In a phase II study of pembrolizumab in colorectal cancer, response rate and immune-related progression-free survival was significantly greater in tumours with mismatch repair deficiency status compared with those without (Le *et al*, 2015). On average, 1782 somatic mutations were identified in mismatch repair-deficient tumours (*n*=9) (by far surpassing the mutational load in melanoma and NSCLC), whereas, on average, only 73 somatic mutations were observed in mismatch repair-proficient tumours (*n*=6) (Le *et al*, 2015). This is predicted to translate into 20 × more mutation-associated neoantigens in mismatch repair-deficient compared with mismatch-proficient tumours (Le *et al*, 2015). In microsatellite unstable endometrial cancer, due to mutations in DNA polymerase epsilon (*POLE*) there is a higher number of CD3+ and CD8+ tumour-infiltrating lymphocytes (TILs), as well as increased PD-1 expression on TILs compared with microsatellite stable tumours, possibly due to increased antigenicity (Howitt *et al*, 2015). Similarly, in a case of hypermutated glioblastoma (GBM) associated with a germline mutation in DNA *POLE*, clinical and immunological response to immune checkpoint inhibition with pembrolizumab has been demonstrated (Johanns *et al*, 2016).

Along with studies demonstrating associations between mutational load and response to immune checkpoint inhibition, a recent study has demonstrated that loss of mutation-associated neoantigens in tumours is associated with resistance to immune checkpoint inhibitor treatment in patients with NSCLC (Anagnostou *et al*, 2016). Interestingly also, intratumoural neoantigen heterogeneity has been shown to affect response to immune checkpoint inhibitors, with higher response rates in tumours predicted to have a high clonal neoantigen burden (McGranahan *et al*, 2016). It is possible that in inherently immunoevasive tumours, significantly increasing mutational load (i.e., antigenicity) lowers the threshold of immunogenicity required to result in responses to immune checkpoint inhibition, and notably, pembrolizumab has recently received accelerated FDA approval for the treatment of microsatellite instability-high (MSI-H) or mismatch repair-deficient (dMMR) tumours, irrespective of tumour type.

Similar to mismatch repair deficiency, defects in other components of the DNA damage response also result in unique mutational signatures in tumours (Alexandrov *et al*, 2013). For example, breast tumours from patients with germline mutations in *BRCA1* or *BRCA2* harbour a greater number of clonal mutations compared with *BRCA1/2* wild-type tumours (Nik-Zainal *et al*, 2012), and in a study of gastric cancer, an association between ATM loss and microsatellite instability has been demonstrated (Kim *et al*, 2014). Whether these observations translate into increased responses to immune checkpoint inhibitors has yet to be demonstrated. Interestingly however, in NSCLC, mutations in DNA repair genes such as *POLD1*, *POLE*, *BRCA2*, *PRKDC*, *MSH2*, *RAD51C*, *LIG3* and *RAD17* were frequently identified in tumours with high mutational burden, the majority of which had a partial response to pembrolizumab (Rizvi *et al*, 2015). Along with having high mutational loads, DNA damage response (DDR)-deficient tumours may also have unique immunological characteristics and at least *BRCA1/2* mutant tumours have been shown to be associated with higher levels of TILs, increased secretion of lymphocyte attractants (eg, C-X-C motif ligand (CXCL) 10 (CXCL10)) and upregulation of immune suppressive ligands such as PD-L1 (Mulligan *et al*, 2013; Strickland *et al*, 2016).

DNA damaging agents are mutagenic, as demonstrated by the increased risk of secondary cancers following treatment with radiotherapy or chemotherapeutics such as etoposide, and also by the mutational signatures associated with some treatments (Alexandrov *et al*, 2013; Murugaesu *et al*, 2015). Inhibition of PARP in sensitive tumour cells, for example those carrying mutations in the *BRCA* gene, results in accumulating levels of DNA damage and genomic instability, ultimately resulting in cell death (Farmer *et al*, 2005). One may extrapolate that in cells that survive, the neoantigen load is likely to rise, thereby diversifying epitopes available for recognition by T cells (epitope spreading) (Jackaman *et al*, 2012). Importantly, the mutagenic potential of DNA damaging agents likely differs across classes of drugs and it cannot be assumed that all chemotherapy will increase neoantigen load in tumours equally. Interestingly, the heterogenous increase in mutations that arise as a result of treatment with DNA damaging drugs such as anthracyclines and platinum-containing regimens, although increasing the subclonal neoantigen population, may not result in the clonal neoantigen presentation required for significant sensitivity to immune checkpoint inhibition (Murugaesu *et al*, 2015; McGranahan *et al*, 2016).

### Increasing antigen presentation

A number of chemotherapies, including gemcitabine, oxaliplatin and cyclophosphamide, have been shown to increase antigen presentation by upregulating MHC class I expression on tumour cells (Liu *et al*, 2010). The same agents have also been shown to promote dendritic cell maturation, priming them for an adaptive immune response (Liu *et al*, 2010). Cyclophosphamide in particular has been shown to expand dendritic cell precursor populations that promotes efficient T-cell priming (Sistigu *et al*, 2011).

### Immunogenic cell death

Neoantigen exposure is insufficient in isolation to stimulate an adaptive immune response (Galluzzi *et al*, 2016). The context for neoantigen exposure seems to be critical, as demonstrated by the fact that many neoantigens with the potential to stimulate T-cell responses in healthy patients go unnoticed in a host with cancer (Strønen *et al*, 2016). Immunogenicity and resulting immunological memory requires antigenicity – exposure of neoantigens, as well as adjuvanticity – the presence of a danger signal that activates the innate immune system (Galluzzi *et al*, 2016). Danger signals from tumours are provided by damage-associated molecular patterns (DAMPs); host molecules that are released from dying cells. Release of DAMPs stimulates the recruitment of APCs to sites of immunogenic cell death (ICD), where they process and present tumour neoantigens, thereby priming an adaptive immune response.

The gold standard for measuring levels of ICD utilise vaccination experiments, whereby murine dying cells are injected into immunocompetent syngeneic mice that are later challenged with living cancer cells of the same type (Kepp *et al*, 2014). Some chemotherapeutics can induce ICD including, for example, anthracyclines (doxorubicin, epirubicin and idarubicin), mitoxantrone, oxaliplatin, cyclophosphamide and bortezomib (Bezu *et al*, 2015). The danger signals or DAMPs released during chemotherapy-induced ICD include: plasma membrane exposure of endoplasmic reticulum chaperones such as calreticulin (CALR), secretion of ATP, release of double-stranded DNA resulting in activation of stimulator of interferon genes (STING) and release of type I interferon and proinflammatory cytokines (Barber, 2015), secretion CXCL10, as well as the release of high-mobility group box 1 (HMGB1) and annexin A1 (ANXA1) (Galluzzi *et al*, 2016). In particular, STING activation appears to be highly relevant to the immune response to DNA damaging agents, whereby DNA accumulation in the cytosol results in type I IFN production due to stimulation of the STING pathway (Kondo *et al*, 2013; Härtlova *et al*, 2015; Erdal *et al*, 2017).

Interestingly, the level of ICD is not necessarily equal across classes of chemotherapeutics; cisplatin, for example, does not induce ICD like oxaliplatin due to a failure to release CALR (Bezu *et al*, 2015). In fact, attenuation of any element of DAMP signalling results in a failure to elicit ICD as has been shown with a number of chemotherapeutics in routine clinical use (Bezu *et al*, 2015). Using combinatorial strategies, it may be possible to restore ICD; for example, ER-stressing agents such as pyridoxine have the ability to render cisplatin treatment immunogenic in preclinical studies (Bezu *et al*, 2015). It is also worth noting that in the context of treatment with *bona fide* immunogenic chemotherapy, to date there does not appear to be any evidence that mutational load affects ICD (Galluzzi *et al*, 2016).

### Changing the cytokine milieu within the TME

As discussed further below, in response to DNA damaging chemotherapy, the cellular DDR coordinates signalling pathways that result in the release of proinflammatory cytokines such as NF-*κ*B and IFN-*α* (Chatzinikolaou *et al*, 2014). The release of cytokines into the extracellular space has a bystander effect on neighbouring cells that results in an immunogenic TME (Malaquin *et al*, 2015). Interestingly, in mice harbouring defects in the nucleotide excision DNA repair pathway (NER), DNA damage leads to chronic autoinflammatory signalling (Karakasilioti *et al*, 2013). Persistent DNA damage results in transcriptional derepression of proinflammatory cytokines such as TNF-*α* and IL-6 in a manner dependent on the apical DDR signalling kinases ataxia telangiectasia mutated (ATM) and ataxia telangiectasia and Rad3-related protein (ATR) (Karakasilioti *et al*, 2013). A similar phenomenon has also been demonstrated following treatment with the PARP inhibitor BMN 673. Treatment of *Brca1*−*/*− mice with BMN 673 resulted in significantly increased levels of IFN-*γ* and TNF-*α*, as well as increased levels of peritoneal CD8+ and natural killer (NK) cells (Huang *et al*, 2015). In preclinical combination studies, CTLA-4 blockade has been shown to synergise with PARP inhibition in *Brca1-*deficient mouse models of ovarian cancer in a manner dependent on IFN-*γ* secretion into the TME (Higuchi *et al*, 2015). CTLA-4 blockade has also been shown to synergise with ixabepilone, etoposide and gemcitabine treatment in preclinical mouse models of cancer, although the mechanism for this synergy has not been fully described (Jure-Kunkel *et al*, 2013). In a study of DDR-deficient breast cancer cells (as defined using a molecular signature of DDR deficiency), DDR deficiency was associated with increased production of chemokines CXCL10 and CCL5, both of which are important for PBMC chemotaxis (Parkes *et al*, 2017). All these studies suggest that generating chronic DNA damage in cancer cells, particularly those deficient in DNA repair, generates a proinflammatory environment and immunogenic tumours.

For many DNA damaging agents, it is difficult to tease apart effects on the immune system that occur indirectly as a result of a DDR-induced ‘stress’ response *vs* those occurring independently of DNA damage. However, some chemotherapies are recognised to be directly immunomodulatory, with cyclophosphamide perhaps being the best example in this regard. Low-dose cyclophosphamide treatment results in higher levels of IFN-*γ* and IL-2, both TH1 cytokines that promote cell-mediated immune activities (Sistigu *et al*, 2011).

### Indirectly lifting immunosuppression: downregulation of MDSCs and Tregs

Regulatory T (Tregs) are essential for the maintenance of self-tolerance, and increasing the numbers of Treg cells in the TME is one mechanism by which tumours evade immunosurveillance (Motz *et al*, 2014). Cyclophosphamide treatment has been shown to enhance the effects of anti-tumour HER-2/neu (neu)-targeted vaccines in *neu*-N mice, at least in part through depleting Treg levels (Ercolini *et al*, 2005). Similarly, in mouse models of glioblastoma, low-dose temozolamide has been shown to result in depletion of the Treg cell population (Banissi *et al*, 2009). Inhibition of the MDSC population by chemotherapy such as gemcitabine and 5-FU may also contribute positively to antitumour immune responses following treatment with DNA damaging agents (Suzuki *et al*, 2005; Vincent *et al*, 2010).

### Effects on PD-1/PD-L1 expression

PD-L1 expression is associated with a poor prognosis across a range of tumour types (Luo and Fu, 2016). Several studies have demonstrated that chemotherapy leads to an upregulation of PD-L1 expression in tumours, and in some cases this has been linked to chemotherapy resistance (Yan *et al*, 2016; Zhang *et al*, 2016). Other studies have reported a downregulation of PD-L1 expression on tumour cells following chemotherapy (Sheng *et al*, 2016) or a redistribution of PD-L1 from the cell surface to nuclear membrane (Ghebeh *et al*, 2010). Common to all these studies is the notion that PD-L1 expression is dynamic and can be affected by DNA damaging agents. Multiple factors are likely to influence PD-L1 expression, however, including type of chemotherapeutic agent, tumour type, baseline PD-L1 expression and response to treatment. The overriding hypothesis is that for several DNA damaging agents, immune-mediated clearance of the tumour contributes to chemosensitivity and blockade of PD-1/PD-L1 signalling may therefore reverse resistance.

In summary, although many DNA damaging agents/immunotherapy combinations might be additive in their antitumour effects, synergy may only be achieved with clear biology-driven combinations that results in ICD and optimal priming of the host immune system, and microenvironment cytokine milieu.

## Enhancing DNA damage using inhibitors of DDR signalling

Following DNA damage in cells, the DDR engages a spectrum of signalling pathways that result in downstream activation of a number of effector processes including DNA repair, cell cycle checkpoint activation and transcriptional regulation, among others (Jackson and Bartek, 2009). Deficiency in the DDR is a hallmark of cancer and germline or somatic mutations in DDR genes can be identified across a range of tumour types (Kandoth *et al*, 2013).

Inhibiting the DDR in tumours is a promising clinical strategy and a number of DDR inhibitors are now in clinical development (Brown *et al*, 2017). DDR inhibitors have the potential to increase mutational burden in tumours, particularly in cancers with high levels of endogenous DNA damage or in combination with exogenous DNA damaging agents. In addition, combining DNA damaging agents with DNA repair inhibitors naturally results in greater and more persistent DNA damage and there is intense interest in how this may promote STING activation and expression of Th1 cytokines (Härtlova *et al*, 2015). Several trials investigating DNA repair inhibition in combination with immune checkpoint inhibition are ongoing (Brown *et al*, 2017) (Table 2), but we must be mindful of the fact that an intact DDR plays an important role in immunity and DDR inhibition has the potential to attenuate rather than stimulate an immune response (Chatzinikolaou *et al*, 2014).

Many key players in the DDR have fundamental roles in innate and adaptive immunity (Ioannidou *et al*, 2016). For example, *Dna-pkcs* knockout mice have severe combined immunodeficiency due to a defect in V(D)J recombination, and ataxia telangiectasia, a syndrome arising due to germline mutations in *ATM*, is characterised in part by an albeit variable immunodeficient phenotype. In fact, it is widely accepted that mechanisms of DNA repair and immunity have evolved in parallel (Ioannidou *et al*, 2016). An intact DDR is essential for proficient innate immune activation, following, for example, the presence of foreign viral DNA in cells and, in particular, results in expression of ligands for the activating NK cell receptor NKG2D as well as release of type I interferons and nuclear factor-*κ*B (NF-*κ*B) that promote antigen presentation (Chatzinikolaou *et al*, 2014; Tang *et al*, 2014). The DDR therefore provides an essential link between the detection of nuclear DNA damage and an appropriate immune response (Ioannidou *et al*, 2016; Nakad and Schumacher, 2016). Given the proinflammatory effects of DDR signalling following DNA damage, inhibiting these processes has the potential to antagonise the effects of immune checkpoint inhibitors. In addition, many key players in the DDR function in multiple cellular processes beyond DNA repair (Blackford and Jackson, 2017) and therefore clinical combination studies require careful consideration, along with appropriate control arms and translational studies to truly test the long-term benefit of combination *vs* monotherapy strategies.

Of the DDR inhibitors in clinical development, PARP inhibitors are most studied and are now licensed for clinical use in ovarian cancer (Brown *et al*, 2016). Along with its role in DNA repair, PARP has a well-established proinflammatory role, and in preclinical models PARP inhibitors attenuate chronic inflammatory and autoimmune conditions in multiple organs (Rosado *et al*, 2013). Recently, it has also been demonstrated that mice deficient for *Parp1* and *Parp2* have a compromised immune response due to defective thymocyte maturation with diminished numbers of peripheral CD4+ and CD8+ T cells (Navarro *et al*, 2017). Treatment of homologous recombination-deficient tumours with PARP inhibitors, particularly those with *BRCA1* or *BRCA1* mutations, generates significant levels of DNA damage however (Farmer *et al*, 2005), and there may be a threshold above which the DNA damage-induced stress signals overwhelm the otherwise anti-inflammatory effects of PARP inhibition. In addition, it is possible although not proven that in the context of synthetic lethality, PARP inhibition is proinflammatory due to overwhelming tumour cell death. In *BRCA1/2* wild-type cells, however, PARP inhibitors may attenuate immune signalling and it will be particularly interesting to determine whether toxicity of immune checkpoint inhibitors in combination with PARP inhibitors is reduced as a result.

## The challenges of combining DNA damaging agents with immune checkpoint inhibitors

### Choice of agent

As detailed in this review, it is clear that DNA damaging agents are not equally immunogenic and therefore choice of combination therapies with immune checkpoint inhibitors needs to be carefully considered. The strategy may also differ depending on the treatment, with agents that result in immunogenic cell death perhaps requiring less or different immune stimulation to those that do not. In *Brca1*-deficient mouse models of ovarian cancer for example, inhibition of CTLA-4 but not PD-1/PD-L1 synergised with PARP inhibitor treatment (Higuchi *et al*, 2015). It is possible that in non-immunogenic tumours, or following treatment with drugs that do not result in ICD, the repertoire of antitumour immune-related responses needs to be broadened, rather than just stimulated (Kvistborg *et al*, 2014).

### Dose

Largely speaking, chemotherapy is employed at the maximum tolerated dose (MTD), where it can be potently myelosuppressive, depleting the immune-related cells we are hoping to stimulate. However, tumour cell death results in the release of neoantigens into the TME as well as the release of ‘danger signals’ that stimulate immunological memory. Carefully designed trials therefore need to consider testing whether maximal tumour cell death (at the MTD) should be compromised in an effort to spare immunoreactive T-cell populations. It is possible that lower doses may offer greater immune modulation; high-dose cyclophosphamide, for example, depletes dendritic cell precursors, whereas lower doses increase dendritic cell pools and promote T-cell priming (Sistigu *et al*, 2011). With respect to stimulation of vaccine responses, low-dose cyclophosphamide has a narrow therapeutic window (Emens *et al*, 2009). Similarly, low-dose temozolamide (TZ) but not high-dose TZ results in depletion of the Treg cell population (Banissi *et al*, 2009). In preclinical studies of tumour-specific vaccines, chemotherapy administration at a dose just above that which starts to cause cytopenias was optimal for enhancing vaccine efficacy, suggesting that dosing just below the MTD may be optimal (Machiels *et al*, 2001).

For DNA damaging compounds that are clearly immunomodulatory beyond their ability to cause ICD, there is some suggestion that metronomic regimens will lend themselves towards combination strategies with immune checkpoint inhibitors. At least for some DNA damaging agents such as gemcitabine, cell death appears important for immunogenicity (Nowak *et al*, 2003). In a study utilising animal models of mesothelioma, gemcitabine lost its immunogenicity on chemoresistant cell lines when apoptosis did not occur (Nowak *et al*, 2003). Assuming in this instance that the immunophenotype of chemoresistant *vs* sensitive tumours are equal (which may not necessarily be the case), cell death was important for tumour-antigen-specific leukocyte proliferation (Nowak *et al*, 2003). Certainly, preclinical and early-phase clinical studies should investigate optimal immune-modulating doses of DNA damaging agents by utilising pharmacodynamic (PD) biomarkers of changes in the tumour immune profile.

### Scheduling and sequencing of combinations

Most if not all anticancer combination therapies are currently administered concurrently, and in the large part the optimal sequencing of agents has not been fully explored. To maximise the efficacy of immune checkpoint inhibitors, it might be advantageous to prime the immune system, administering DNA damaging agents up front, and data from a number of studies now support this. Administration of DTIC 1 day before vaccination with a combination of gp100 and melanoma-specific antigen vaccine resulted in a significantly improved long-lasting memory CD8+ T-cell response compared with vaccine alone (Nisticò *et al*, 2009). In a phase II trial of carboplatin and paclitaxel (carbo/taxol)±concurrent or phased ipilimumab (ipi), only phased treatment (carbo/taxol for 2 cycles followed by carbo/taxol/ipi for 4 cycles) showed an improved immune-related (ir) PFS benefit over chemotherapy alone and a trend towards an OS benefit in this arm, in patients with small-cell lung cancer (SCLC) (Reck *et al*, 2013). In this study, there was no benefit between the arms when considering a nonimmune-related PFS end point (Reck *et al*, 2013). However, in a similar study in NSCLC, carbo/taxol plus phased ipilimumab demonstrated a modest improvement in irPFS and PFS over chemotherapy alone (Lynch *et al*, 2012). In a mouse model of mesothelioma, concurrent administration of anti-CTLA-4 blocking antibody and gemcitabine was superior in terms of overall survival compared with sequential administration of either anti-CTLA antibody or gemcitabine first (Lesterhuis *et al*, 2013). These studies suggest that upfront treatment with chemotherapy followed by a period of concurrent treatment with chemotherapy and immune checkpoint inhibition might be optimal, but further studies testing proof-of-concept data and incorporating PD end points are required in order to truly establish the optimal scheduling in the clinic. Testing the immunological effects of chemotherapy combinations in murine studies might not provide all the answers, but perhaps it should be a simple precursor to strengthen the scientific rationale of a large and costly clinical trial. Similarly, a case could be made for randomised biomarker proof-of-concept phase 2 trials to guide scheduling and immunomonitoring before embarking on phase 3 studies, although knowing that endless combinations and schedules could potentially be tested.

### Toxicity

Published and presented data from clinical trials combining DNA damaging chemotherapy and immune checkpoint inhibitors suggests that these agents can be safely combined. Given the non-overlapping toxicity of DNA damaging chemotherapy and immune checkpoint inhibition monotherapy, combination trials have been able to achieve optimal doses of both agents. Foreseeable challenges surround the practicalities of delivering both agents however. Many DNA damaging chemotherapy regimens incorporate significant doses of corticosteroids, either to limit hypersensitivity reactions or as part of the anti-emetic regimen. The immunosuppressive effects of steroids have the potential to attenuate the effects of the immune checkpoint inhibitors, although there is limited and inconclusive evidence to determine how detrimental steroid use will be on overall efficacy. For those symptoms that do overlap, such as diarrhoea, fatigue and myalgias, determining the likely causative agent will be challenging and will have significant implications on the overall management. In particular, oncologists will be nervous about reducing the dose intensity of chemotherapy, particularly in an adjuvant or neoadjuvant setting, that might be difficult to avoid when managing concurrent immune-related toxicities.

## Combining DNA damaging agents and immunotherapy in the clinic

**Figure 2:**
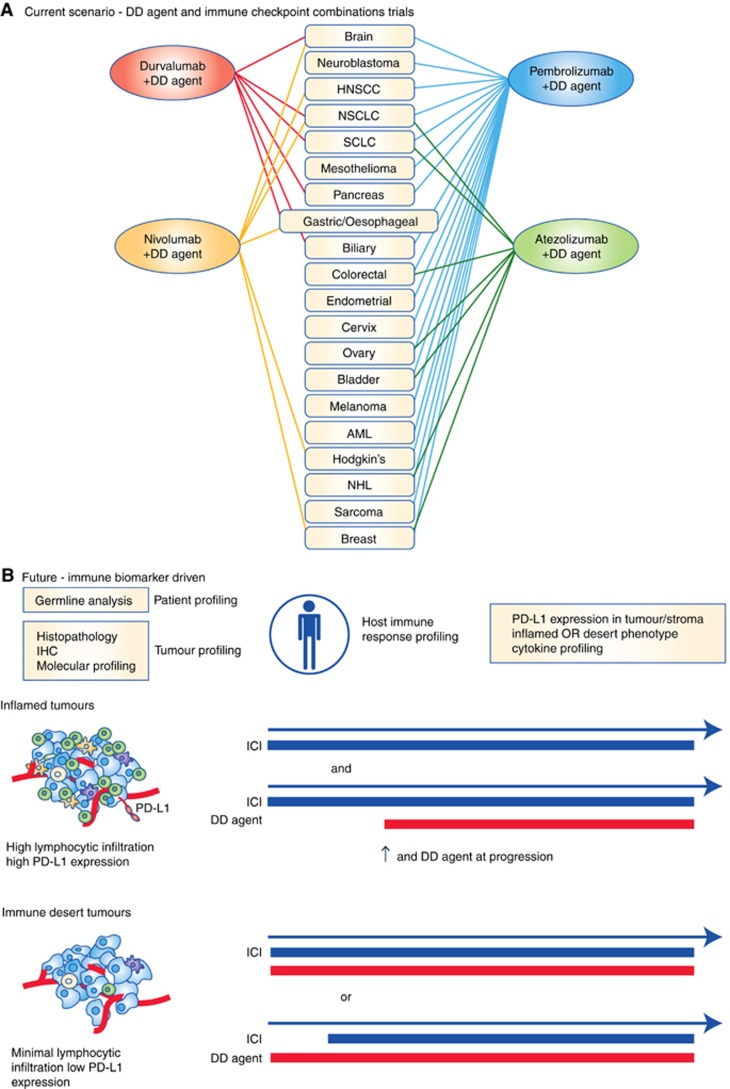
**Combination strategies for DNA damaging therapeutics and immunotherapy.** (**A**) Chemotherapy combination trials with current PD-1 and PD-L1 checkpoint inhibitors as registered with www.clinicaltrials.gov. AML=acute myeloid leukaemia; DD agent=DNA damaging agent; HNSCC=head and neck squamous cell cancer; NHL=non-Hodgkin’s lymphoma; NSCLC=non-small-cell lung cancer; SCLC=small-cell lung cancer. (**B**) Proposed biomarker-driven approach to chemotherapy/immunotherapy combination trials. Multiple biomarkers that incorporate profiling of the tumour, patient and host immune response combined to determine tumour immune phenotype (Blank *et al*, 2016; Hegde *et al*, 2016). Inflamed tumours might demonstrate high levels of effector T cells (green), APCs (orange) and MDSCs (purple), with low PD-L1 expression and may respond to immune checkpoint inhibitor (ICI) monotherapy, requiring combination treatment with DNA damaging (DD) agents on progression only. Compare with the reverse in immune desert tumours that may require priming with DD agents followed by concurrent treatment with an ICI.

There are now >200 clinical trials listed on clinicaltrials.gov that are testing immune checkpoint inhibitors in combination with DNA damaging chemotherapies (Figure 2A). Between the four anti-PD-1/PD-L1 agents that are most advanced in terms of clinical development, it would appear that every standard of care chemotherapy regimen in every tumour type is being tested in combination with at least one immune checkpoint inhibitor (Figure 2A). There has been an almost exponential increase in the number of immunotherapy/chemotherapy trials being conducted over the past 12–24 months and only time will tell whether in being so hasty, the scramble to registration will truly pay off. To our knowledge, of those trials that are published or that have preliminary data available, combination treatment with PD-1/PD-L1 inhibitors and DNA damaging chemotherapy certainly has the potential to be superior to chemotherapy alone (Harris *et al*, 2016; Langer *et al*, 2016). We have yet to see an immunotherapy monotherapy control arm however and therefore it is difficult to determine the proportion of patients who are truly benefiting from the combination. Equally, it is too early to be certain of whether long-term survival benefit is improved using combination treatments upfront. With regards to immunotherapy/DDR inhibitor combination studies, a phase I trial of Durvalumab (PD-L1 inhibitor) in combination with olaparib (PARP inhibitor) has shown promising antitumour activity, with the combination proving to be safe, although haematological toxicity was observed more frequently compared with historical olaparib monotherapy studies (Lee *et al*, 2017). Further studies to evaluate the clinical effectiveness, as well as translation work to understand the synergy of this combination, will be of great interest.

Immunotherapy biomarkers have been extensively reviewed previously and a detailed discussion here is beyond the scope of this review (Gibney *et al*, 2016). Needless to say however, utilising PD biomarkers should be a compulsory component of early-phase combination studies in order to determine optimal doses and scheduling – in particular, identifying robust biomarkers of ICD and cytokine signatures of immune activation. Equally, determining early biomarkers of response should be incorporated into all trials, as effective patient selection will maximise efficacy and will also facilitate decision making regarding continuation of treatment (Lesterhuis *et al*, 2017).

## Conclusion

Tumour immunobiology is complex and the extensive network of overlapping mechanisms utilised by tumours to evade immunosurveillance makes optimally targeting this process a considerable challenge. Combining DNA damaging chemotherapy with immune checkpoint inhibitors has the potential to reverse many of these immunoevasive strategies. Many unanswered questions remain however, including choosing the optimal agents, determining effective doses and schedules and managing toxicity. Establishing clinically measurable pharmacodynamic biomarkers, as well as robust biomarkers of response to combination treatments, is going to be essential.

Although DNA damaging chemotherapy undeniably has the potential to synergise with immune checkpoint inhibitors in the clinic, the scientific rationale is not immediately obvious in many ongoing clinical studies. Many chemotherapy/immunotherapy combinations are entering late-phase clinical studies following only small safety-orientated phase I trials, with limited or absent investigation of appropriate PD biomarkers. As we are discovering, there is a sliding scale of immunogenicity within tumours (Blank *et al*, 2016; Kingwell, 2016). At one end of the scale, ‘inflammatory’ tumours may need minimal immune stimulation, requiring combination strategies only upon resistance to immune checkpoint inhibition. At the other end of the spectrum, an ‘immune desert’ designates tumours that are likely to require immunological priming in conjunction with maximal immune stimulation to see benefit (Figure 2B). It is essential that we design trials that incorporate not only patient selection biomarkers, but also pharmacodynamic biomarkers that consolidate our understanding of the biology, confirm or refute our hypotheses and result in the optimal combinations in the optimal sequence and at the optimal doses. Race to registration is threatening a careful and considered approach that has the risks of never realising the true potential of these combinations.

## Figures and Tables

**Table 1:** Immune checkpoint inhibitors with a licence for use in cancer

	**Target**	**Tumour group**	**Line of treatment**	**Trial name**	**Regulatory approval**			**Citation**
					**FDA**	**EMA**	**NICE**	
Ipilimumab	CTLA-4	Melanoma	2nd	NA	Mar 11	May 11	Dec 12	PMID: 20525992
			1st	NA	NA	Sep 13	Jul 14	PMID: 21639810
			Adjuvant	EORTC 18071	Oct 15	NA	NA	PMID: 27717298
Nivolumab	PD-1	Melanoma	2nd	Checkmate 037	Dec 14	Apr 15	Feb 16	PMID: 25795410
			1st	Checkmate 066	NA	Apr 15	Feb 16	PMID: 25399552
		Squamous lung	2nd	Checkmate 017	Mar 15	Sep 15	NA	PMID: 26028407
		Nonsquamous lung	2nd	Checkmate 057	Oct 15	Sep 15	NA	PMID: 26412456
		RCC	2nd	Checkmate 025	Nov 15	Feb 16	Nov 16	PMID: 26406148
		Hodgkin’s lymphoma	3rd	NA	May 16	Oct 16	NA	PMID: 27451390
		Head and neck	2nd	Checkmate 141	Nov 10	May 17	NA	PMID: 27718784
		Urothelial	2nd	Checkmate 275	Feb 17	Jun 17	NA	PMID: 28131785
Pembrolizumab		Lung	2nd	Keynote 001	Oct 15	Jun 16	Dec-16	PMID: 25891174
			1st	Keynote 024	Oct 16	Dec 16	NA	PMID: 27718847
			1st +Carbo/Pem	Keynote 021	May 17	NA	NA	PMID: 27745820
		Melanoma	2nd	Keynote 001	Sep 14	May 15	Oct-15	PMID: 25034862
		Head and neck	2nd	Keynote 012	Aug 16	NA	NA	PMID: 27247226
		Hodgkin’s lymphoma	2nd	Keynote 013 + 087	Mar 17	May 17	NA	PMID: 28441111
		Urothelial	1st (platinum ineligible)	Keynote 052	May 17	NA	NA	
			2nd	Keynote 045	May 17	NA	NA	PMID: 28212060
		MSI-H/dMMR solid tumour	2nd	NA	May 17	NA	NA	
Atezolizumab	PD-L1	Urothelial	2nd	NA	May 16	Jul-17	NA	PMID: 26952546
			1st (platinum ineligible)	IMvigor210	Apr 17	Jul 17	NA	
		Lung	2nd	OAK	Oct 16	Jul 17	NA	PMID: 27979383
Durvalumab		Urothelial	2nd	Study 1108	May 17	NA	NA	PMID: 27269937

Abbreviations: CTLA-4=cytotoxic T-lymphocyte-associated protein 4; dMMR=mismatch repair deficient; EMA=European Medicines Agency; FDA=Food and Drug Administration; MSI-H=microsatellite instability high; NA=not available; NICE=National Institute for Health and Care Excellence; PD-1=programmed cell death-1; PD-L1=programmed cell death ligand-1; RCC=renal cell carcinoma.

**Table 2:** Ongoing combination trials with DDR and immune checkpoint inhibitors (www.clinicaltrials.gov)

**ICI**	**Tumour group**	**Target population**	**DDR agent**	**Phase**	**Arms**	**Planned n**	**Trial status**	**NCT**	**Citation/remarks**
Durvalumab	Breast	3rd line	Olaparib	1/2	Olaparib + Durvalumab	133	Recruiting	NCT02734004	
	Gastric	2nd line							
	Ovarian	Platinum sensitive							
	SCLC	2nd line							
	NSCLC/ SCLC	2nd or higher line	Olaparib	1/2	Durvalumab + Olaparib	338	Recruiting	NCT02484404	
	Breast	TNBC, < 3 prior lines			Durvalumab + Cediranib				
	Ovarian	Platinum resistant			Durvalumab + Olaparib + Cediranib				
	Colorectal	3rd line							
	Prostate	mCRPC							
	Ovary	gBRCA	Olaparib	1/2	Olaparib + Durvalumab + Tremelimumab	39	Not yet recruiting	NCT02953457	
	NSCLC	Refractory	AZD6738	1	AZD6738 + Durvalumab	114	Recruiting	NCT02264678	Has other arms involving AZD 6738 with other agents
	HNSCC								
Tremelimumab	Ovarian	2nd line +	Olaparib	1/2	Tremelimumab + Olaparib	50	Recruiting	NCT02571725	gBRCA only
Pembrolizumab	Breast	Up to 3 prior lines	Niraparib	1/2	niraparib + pembrolizumab	114	Recruiting	NCT02657889	TNBC only
	Ovarian	Up to 4 prior lines							Platinum resistant/refractory only
Nivolumab	NSCLC	1st line metastatic	Carboplatin + paclitaxel or pemetrexed + Veliparib	2	Veliparib + nivolumab + platinum doublet chemotherapy	184	Recruiting	NCT02944396	NA
					Veliparib + platinum doublet chemotherapy				
	Adv solid tumours	Refractory to std therapy	Veliparib	1	Veliparib + Nivolumab	50	Not yet recruiting	NCT03061188	
Atezolizumab	Breast	Any prior therapy allowed	Veliparib	2	Veliparib	90	Recruiting	NCT02849496	TNBC + gBRCA only
					Atezolizumab				
					veliparib + atezolizumab				
BGB-A317	Adv solid tumours	2nd line +	BGB-290	1	BGB-A317 + BGB-290	124	Recruiting	NCT02660034	

Abbreviations: DDR=DNA damage response; gBRCA=germline BRCA; HNSCC=head and neck squamous cell cancer; ICI=immune checkpoint inhibitor; mCRPC=metastatic castration-resistant prostate cancer; NA=not available; NSCLC=non-small-cell lung cancer; SCLC=small-cell lung cancer; TNBC=triple-negative breast cancer.

## Box 1 - Mechanisms by which tumours evade immunosurveillance

**Restricting T-cell accumulation at tumour sites**
  - The cellular composite of the tumour microenvironment is unique compared with normal stromal tissue, comprising high levels of cancer-associated fibroblasts, myeloid-derived suppressor cells (MDSCs) and tumour-associated macrophages. Nitric oxide production by MDSCs within the TME results in nitration of the chemokine CCL2 to N-CCL2 that traps T cells in the TME, while serving as a monocyte chemoattractant (Molon *et al*, 2011). In addition, epigenetic silencing of chemokines CXCL9 and CXCL10 inhibits T-cell trafficking to tumours (Peng *et al*, 2015).
**Promoting T-cell tolerance**
  - ***Effects of the tumour vasculature:*** Immune suppressive Treg cells are relatively protected against Fas ligand-mediated cell killing due to having high expression of the apoptosis inhibitor c-FLIP (Motz *et al*, 2014). High levels of endothelial Fas ligand (FasL) in tumours, as a result of tumour-derived vascular endothelial growth factor A (VEGF-A), interleukin 10 (IL-10) and prostaglandin E2 (PGE2), causes disproportionately high levels of Treg compared with CD8+ T-cell populations at tumour sites (Motz *et al*, 2014).
  - ***Indole 2,3-dioxygenase (IDO):*** The TME can directly inhibit T-cell expansion through the production of IDO that can be constitutively and inducibly expressed by dendritic cells (DCs), MDSCs and cancer cells themselves in response to inflammation (Munn and Mellor, 2013). Indole 2,3-dioxygenase (IDO) catabolises tryptophan to kynurenine, and tryptophan depletion with resulting kynurenine accumulation inhibits T-cell proliferation (Munn and Mellor, 2013). IDO also promotes the conversion of naive T cells to Treg cells, increases IL-6 expression that augments MDSC functions and blocks the reprogramming of Tregs to helper-like cells (Munn and Mellor, 2016). Overall therefore, IDO is important for both controlling inflammation by pushing APCs towards an immunosuppressive phenotype and creating acquired antigen-specific tolerance in T cells (Munn and Mellor, 2016).
**Inhibiting T-cell activation at tumour sites**
  - ***Upregulation of PD-L1 expression by tumours:*** This contributes to local suppression of T-cell activation and evasion of immunosurveillance. There is now good clinical evidence that tumours with high PD-L1 expression are more likely to respond to PD-1 or PD-L1 inhibitor monotherapy such as pembrolizumab and atezolizumab (Herbst *et al*, 2014; Garon *et al*, 2015); however, the negative predictive value of PD-L1 expression is not 100% and several other important biomarkers of response are likely to exist.
  - ***Defects in antigen processing and presentation***: This includes downregulation of MHC class I on tumour cells, which is also a common mechanism by which tumours avoid CD8+ T-cell recognition (Reeves and James, 2017).
